# Shared Genetics of Multiple System Atrophy and Inflammatory Bowel Disease

**DOI:** 10.1002/mds.28338

**Published:** 2020-10-27

**Authors:** Alexey A. Shadrin, Sören Mucha, David Ellinghaus, Mary B. Makarious, Cornelis Blauwendraat, Ashwin A.K. Sreelatha, Antonio Heras-Garvin, Jinhui Ding, Monia Hammer, Alexandra Foubert-Samier, Wassilios G. Meissner, Olivier Rascol, Anne Pavy-Le Traon, Oleksandr Frei, Kevin S. O’Connell, Shahram Bahrami, Stefan Schreiber, Wolfgang Lieb, Martina Müller-Nurasyid, Ulf Schminke, Georg Homuth, Carsten O. Schmidt, Markus M. Nöthen, Per Hoffmann, Christian Gieger, Gregor Wenning, European Multiple System Atrophy Study Group, J. Raphael Gibbs, Andre Franke, John Hardy, Nadia Stefanova, Thomas Gasser, Andrew Singleton, Henry Houlden, Sonja W. Scholz, Ole A. Andreassen, Manu Sharma

**Affiliations:** 1 NORMENT, Institute of Clinical Medicine, University of Oslo and Division of Mental Health and Addiction, Oslo University Hospital, Oslo, Norway; 2 Institute of Clinical Molecular Biology, Christian-Albrechts-University of Kiel, Kiel, Germany; 3 Neurodegeneratlve Diseases Research Unit, National Institute of Neurological Disorders and, Stroke, National Institutes of Health, Bethesda, Maryland, USA; 4 Laboratory of Neurogenetics, National Institute on Aging, National Institutes of Health, Bethesda, Maryland, USA; 5 Centre for Genetic Epidemiology, Institute for Clinical Epidemiology and Applied Biometry, University of Tübingen, Tübingen, Germany; 6 Department of Neurology, Medical University of Innsbruck, Innsbruck, Austria; 7 Service de Neurologie, CRMR Atrophie Multisystématisée, CHU Bordeaux, Bordeaux, France; 8 Inserm, UMR1219, Bordeaux Population Health Research Center, Bordeaux University, ISPED, Bordeaux, France; 9 Univ. de Bordeaux, Institut des Maladies Neurodégénératives, UMR 5293, CNRS, Bordeaux, France; 10 Centre de Reference Maladie Rare Atrophie MultiSystématisée, Centre d’Investigation, Clinique CIC 1436, Services de Pharmacologie Clinique et Neurosciences, NeuroToul COEN Center, Toulouse, France; 11 Centre Hospitalo-Universitaire de Toulouse, 3, INSERM, Toulouse, France; 12 Neurology Department, French Reference Centre for MSA, University Hospital of Toulouse and INSERM U 1048, Institute of Cardiovascular and Metabolic Diseases, Toulouse, France; 13 First Medical Department, University Hospital Schleswig-Holstein, Kiel, Germany; 14 Institute of Epidemiology and Biobank PopGen, Christian-Albrechts-University of Kiel, Kiel, Germany; 15 Institute of Genetic Epidemiology, Helmholtz Zentrum München - German Research Center for Environmental Health, Neuherberg, Germany; 16 Chair of Genetic Epidemiology, IBE, Faculty of Medicine, Ludwig-Maximilians-University (LMU) Munich, Munich, Germany; 17 Department of Internal Medicine I (Cardiology), Hospital of the Ludwig-Maximilians-University (LMU) Munich, Munich, Germany; 18 Department of Neurology, University Medicine Greifswald, Greifswald, Germany; 19 Department of Functional Genomics, Interfaculty Institute for Genetics and Functional Genomics, University Medicine and Ernst-Moritz-Arndt-University Greifswald, Greifswald, Germany; 20 Institute for Community Medicine, Study of Health in Pomerania/KEF, University Medicine Greifswald, Greifswald, Germany; 21 Institute of Human Genetics, University of Bonn, Bonn, Germany; 22 Research Unit of Molecular Epidemiology, Institute of Epidemiology, Helmholtz Zentrum München-German Research Center for Environmental Health, Neuherberg, Germany; 23 Rita Lila Weston Institute, University College London, London, UK; 24 German Center for Neurodegenerative Diseases (DZNE), Tübingen, Germany; 25 Department of Neurodegenerative Diseases, Hertie Institute for Clinical Brain Research, University of Tübingen, Tübingen, Germany; 26 Department of Neurology, Johns Hopkins University Medical Center, Baltimore, Maryland, USA

**Keywords:** multiple system atrophy, inflammatory bowel disease, genetic overlap, conjunctional false discovery rate

## Abstract

**Background:**

Multiple system atrophy (MSA) is a rare neurodegenerative disease characterized by intracellular accumulations of α-synuclein and nerve cell loss in striatonigral and olivopontocerebellar structures. Epidemiological and clinical studies have reported potential involvement of autoimmune mechanisms in MSA pathogenesis. However, genetic etiology of this interaction remains unknown. We aimed to investigate genetic overlap between MSA and 7 autoimmune diseases and to identify shared genetic loci.

**Methods:**

Genome-wide association study summary statistics of MSA and 7 autoimmune diseases were combined in cross-trait conjunctional false discovery rate analysis to explore overlapping genetic background. Expression of selected candidate genes was compared in transgenic MSA mice and wild-type mice. Genetic variability of candidate genes was further investigated using independent whole-exome genotyping data from large cohorts of MSA and autoimmune disease patients and healthy controls.

**Results:**

We observed substantial polygenic overlap between MSA and inflammatory bowel disease and identified 3 shared genetic loci with leading variants upstream of the *DENND1B* and *RSP04* genes, and in intron of the *C7* gene. Further, the *C7* gene showed significantly dysregulated expression in the degenerating midbrain of transgenic MSA mice compared with wild-type mice and had elevated burden of protein-coding variants in independent MSA and inflammatory bowel disease cohorts.

**Conclusion:**

Our study provides evidence of shared genetic etiology between MSA and inflammatory bowel disease with an important role of the *C7* gene in both phenotypes, with the implication of immune and gut dysfunction in MSA pathophysiology.

A growing number of studies suggest that complex diseases often have a highly polygenic structure with shared genetic background.^1,2^. Genome-wide association studies (GWASs) have identified novel loci for many complex diseases.^2^ Polygenic architecture and ubiquitous genetic overlap between complex diseases promote the use of analytical approaches that are designed to exploit these factors and can leverage abundant GWAS data to identify genetic loci shared between diseases.^3^ Polygenic overlap has already been successfully exploited to identify shared genetic loci for various complex diseases, including neurological diseases^4,5^ and psychiatric diseases.^6^

Multiple system atrophy (MSA) is a rare adult-onset progressive neurodegenerative disorder with complex etiology characterized by a combination of parkinsonism, autonomic, cerebellar, or pyramidal signs.^7^ Because of the involvement of α-synuclein, MSA is categorized as a synucleinopathy along with Parkinson’s disease (PD) and dementia with Lewy bodies. Pathologically, it is defined by neuronal cell loss, gliosis, and α-synuclein–positive oligodendroglial cytoplasmic inclusions (GCIs). Neuronal loss in MSA affects striatonigral and olivopontocerebellar structures, and the degree of neuronal loss and GCI density has shown a positive correlation between both lesions, suggesting that the accumulation of GCIs is an important factor in neuronal death in MSA.^8^

The role of immune dysfunction in neurodegenerative diseases has long been debated. For example, one of the earlier studies using autopsy-confirmed PD cases showed a higher level of microglial activation within the substantia nigra, and this increase in microglial activity was identified by human leukocyte antigen (HLA) DR.^9^ In the past decade GWASs have started to reveal the extent of the immune component in the etiopathogenesis of neurodegenerative diseases.^10^ Previously published GWASs and follow-up studies in PD provided unequivocal evidence regarding the role of the HLA region in PD pathogenesis.^10^ A recently published study identified α-synuclein-derived peptides, which regulate the expression of the HLA locus.^11^ Likewise, GWAS studies in tauopathies, a group of diseases such as Alzheimer’s disease (AD), frontotemporal dementia, and progressive supranuclear palsy characterized by an abnormal accumulation of neurofibrillary tangles, have established the role of the immune component, thus lending further support to the notion that immune dysfunction is one of the central pathways to neurodegenerative disorders.^12^

In contrast to other more common neurodegenerative disorders, genetic studies including GWASs have failed to identify disease-associated genetic loci for MSA, although heritability estimates have established a small genetic component in MSA.^13^ Nevertheless, emerging evidence points toward deregulation of mitochondria, energy homeostasis, oxidative stress, and immune dysfunction that could contribute to the α-synuclein inclusions observed in oligodendrocytes in MSA.^14^ Despite this, developing a consensus on potential mechanisms that explain the causes of MSA remains challenging. In the present study, we applied a genome-wide genetic-pleiotropy-informed approach to identify shared genetic risk factors between MSA and autoimmune diseases, with subsequent validation in MSA transgenic mice. Major steps of the study are presented in Figure 1.

## Methods

### Participant Samples

We used GWAS summary statistics on 7 autoimmune disease: inflammatory bowel disease (25,042/34,915 cases/controls),^15^ Crohn’s disease (12,194/34,915),^15^ ulcerative colitis (12,366/34,915),^15^ diabetes mellitus type 1 (7514/9045),^16^ celiac disease (4533/10,750),^17^ rheumatoid arthritis (29,880/73,758),^18^ multiple sclerosis (9772/17,376), and independent GWAS on MSA (918/3864).^19^ MSA cases were clinically diagnosed with possible or probably MSA (n = 699) by movement disorders specialists or pathologically with definite MSA (n = 331) by neuropathologists, according to Gilman criteria. Complete description of the MSA cohort composition and demographic characteristics have been described elsewhere.^19^ Inflammatory bowel disease (IBD), Crohn’s disease (CD), and ulcerative colitis CWASs^15^ were conducted with the same set of controls. IBD cases used in de Lange et al^15^ include CD, ulcerative colitis, and unclassified cases. Details of the inclusion criteria and phenotype characteristics of the GWASs are described in the original publications.

### Genetic Overlap Between MSA and Autoimmune Diseases

To visually assess enrichment of cross-phenotype polygenic overlap between MSA and autoimmune diseases, we generated conditional quantile-quantile (Q-Q) plots^6^ by conditioning MSA on each of 7 autoimmune phenotypes and vice versa. Q-Q plots are commonly used for visualization of *P* values from GWAS summary statistics to assess enrichment of association by plotting quantiles of the observed distribution of association *P* values with the phenotype against quantiles of the *P*-value distribution expected under no association (standard uniform distribution). In the absence of association, the Q-Q plot represents a straight line (diagonal of the first quadrant), whereas deflection from the line indicates the presence of a systematic association. Conditional Q-Q plots are generated by defining subsets of variants based on significance levels in the conditional phenotype and constructing Q-Q plots for associated values in the primary phenotype for each subset separately. Enrichment of genetic overlap between primary and conditional phenotype emerges in the conditional Q-Q plot as successive leftward deflections as the significance with the conditional phenotype increases.

Prior to the construction of Q-Q plots and subsequent analysis of the shared genetic factors, the genomic inflation control procedure described in Andreassen et al^6^ was applied to correct GWAS *P* values for all analyzed phenotypes. In addition, to correct for inflation induced by the linkage disequilibrium (LD), the contribution of each single-nucleotide polymorphism (SNP) was measured considering LD structure in the surrounding region (±20-megabase-pair window), using 100 iterations of random pruning at LD threshold *r*^2^ = 0.1. LD structure (*r*^2^ values) was estimated with PLINK^20^ using the 1000 Genomes Project phase 3 European data.^21^ At each iteration, a set of nearly LD-independent SNPs was selected by taking 1 random SNP in each LD-independent region (clump of SNPs in LD, *r*^2^ > 0.1). The final contribution of each SNP was estimated as an average across all iterations. Because of the underlying complex LD structure encompassing the HLA and the microtubule-associated protein tau regions, which may inflate a conditional Q-Q plot and bias other downstream analyses, SNPs within these regions (hg19 locations chr6:25119106–33,854,733 and chr17:40000000–47,000,000, respectively) were excluded from all presented statistical analyses.

To further characterize polygenic overlap between analyzed phenotypes, we estimated genetic correlations between MSA and 7 autoimmune diseases using LD score regression.^22^

### Shared Loci Between MSA and IBD

The phenotypes that showed substantial genetic overlap with MSA in conditional Q-Q plots were further analyzed with conjunctional false discovery rate (conjFDR) method^6,23^ to identify shared genetic loci between MSA and autoimmune diseases. The conjFDR method combines summary statistics from 2 phenotypes to identify genetic loci associated with both phenotypes simultaneously. In the presence of genetic overlap between analyzed phenotypes, the conjFDR approach offers increased statistical power to discover genetic loci shared between analyzed phenotypes compared with conventional multiple-testing approaches.^24^ The method was effectively applied to discover shared genetic loci in various complex disorders.^5,25^

### Functional Annotation of Identified Loci

Two types of analyses were performed. First, positional and functional annotation of lead variants identified in the conjFDR analyses was performed using FUMA.^26^ Lead variants were annotated with Combined Annotation Dependent Depletion (CADD) scores, measuring the degree of variant deleteriousness on protein structure/function, RegulomeDB scores predict the likelihood of regulatory functionality, and chromatin states estimate transcription/regulatory effects from chromatin states at the locus. Lead variants were queried for known expression quantitative trait loci (cQTLs) in the GTEx portal.^27^ In addition, we scanned blood^28^ (N > 30,000) and brain^29^ (N > 520) eQTL summary statistics with substantially larger sample sizes than corresponding tissues in GTEx (N_blood_ < 400, N_brain_ < 200). LocusCompare^30^ was then applied to check whether loci identified in conjFDR analysis colocalizes with eQTL signal. Although LocusCompare can be useful for visual assessment of the colocalization between conjFDR and eQTL signals, it cannot quantify contributions of specific SNPs to colocalization. In contrast, tools like eCAVIAR,^31^ COLOC,^32^ or SMR^33^ can measure contributions of specific variants and therefore could he more informative. However, these tools require as an input association *P* values, which should follow standard uniform distribution under assumption of no association. This requirement does not hold for FDR values produced by the conjFDR analysis.

### Gene-Level Association Analysis

Exome-wide genotyping data from independent IBD, CD, and MSA exome cohorts of European ancestry were used to assess whether candidate genes selected based on functional annotation of the loci identified in the conjFDR analysis are enriched with disease-associated variants. Neither of these cohorts overlaps with the corresponding GWAS samples. Both IBD (15,236/34,668 cases/controls) and CD (4989/16,307) cohorts were genotyped using an exome chip covering 205,313 common and rare variants in approximately 16,000 protein-coding genes. Whole-exome sequencing data for MSA cohort (358/1297 cases/controls) were generated using the SureSelect Exome target enrichment technology. Individuals of non-European ancestry were removed from the analyses. SKAT test^34^ in all 3 cohorts was conducted using all coding variants within identified genes. *P* values obtained from these tests were corrected for multiple testing using Bonferroni correction.

### Expression Analysis of Candidate Genes in the Degenerating Midbrain of Transgenic MSA Mice

PLP-hαSyn transgenic mice (also called MSA mice^35^) and wild-type controls were kept under temperature-controlled pathogen-free conditions on a light/dark 12-hour cycle. All experiments were performed according to the ethical guidelines of the EU (Directive 2010/63/EU for animal experiments) and the Austrian Federal Ministry of Science and Research (permission BMFWF-66.011/0018-WE/v/3b/2015). Twelve-month-old male mice (5 PLP-hαSyn and 5 wild-type mice) were perfused intracardially with phosphate-buffered saline (pH 74; Sigma) under deep thiopental anesthesia. Brains were extracted, and midbrains were quickly dissected and snap-frozen in liquid nitrogen. Samples were stored at −80°C until further processing. Samples were homogenized in TRIzol reagent (Life technologies) with ULTRA-TURRAX T-8 basic tissueruptor (IKA), and RNA was isolated following the manufacturer’s instructions. RNA samples were retrotranscribed into cDNA using a High-Capacity cDNA Reverse Transcription Kit (Applied-Biosystems). Analyses were performed in a CEX96 Touch Real-Time PCR Detection System (Bio-Rad) using iTaq universal probes supermix (Bio-Rad). *Gapdh* mRNA levels were used as an internal normalization control. All statistical analyses were conducted using the software Graph-Pad Prism 7 (Graphpad Software). The mean ± standard effort of the mean was used to present the results. Comparisons were performed with multiple *t* tests with Bonferroni-Dunn correction. An adjusted *P* < 0.05 was considered statistically significant.

### Identification of Relevant Tissues and Cell Types

The LD-SEG method^36^ was used to determine potential enrichment of tissue-/cell-type-specific categories in MSA heritability. The method was applied to 7 publicly available data sets with tissue-/cell-type-specific gene expression and chromatin state used in the original publication.^36^

### Gene Set Enrichment Analysis

DEPICT^37^ was applied to identify pathways enriched with variants shared between MSA and IBD/CD, as identified in the conjFDR analyses with relaxed significance threshold (conjFDR < 0.35); see Table S6. Additional details of performed analyses are presented in Appendix S1.

## Results

### Genetic Overlap Between MSA and Autoimmune Diseases

**FIG. 2.**
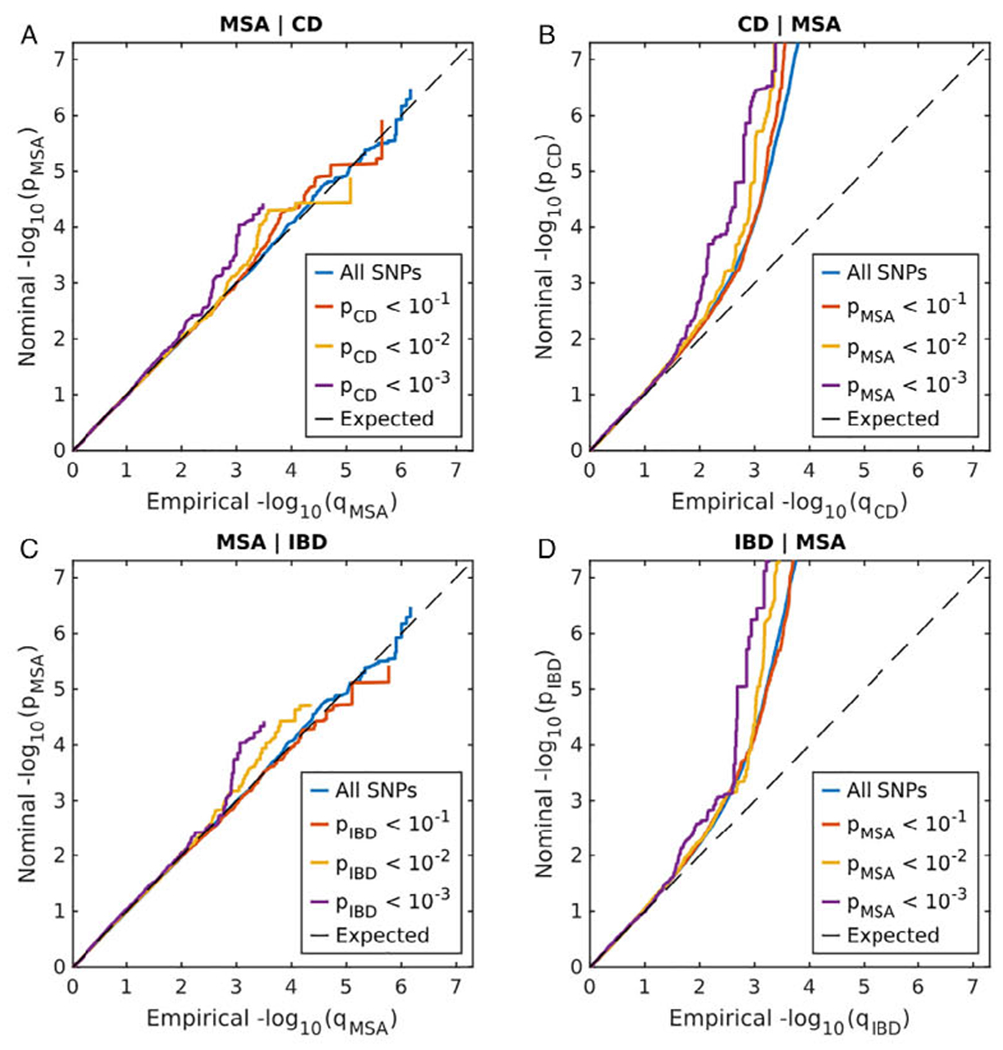
Conditional Q-Q plot showing the relation between expected (*x* axis) and observed (*y* axis) significance of SNPs in the primary phenotype when markers are stratified by their *P* values in the conditional phenotype. A sequence of 4 nested strata is presented: blue, all SNPs; orange, *P*_conditional_phenotype_ < 0.1; yellow, *P*_conditional_phenotype_ < 0.01; and purple, *P*_conditional_phenotype_ < 0.001. Dashed black line demonstrates expected behavior under no association. The increasing degree of leftward deflection from the no-association line for strata of SNPs with higher significance in the conditional phenotype indicates putative polygenic overlap. (**A**) MSA conditioned on CD; (**B**) CD conditioned on MSA; (**C**) MSA conditioned on IBD; (**D**) IBD conditioned on MSA. [Color figure can be viewed at wileyonlinelibrary.com]

Conditional Q-Q plots for association *P* values of MSA and autoimmune diseases showed strong enrichment for CD and IBD (including CD, ulcerative colitis and unclassified IBD cases); see Figure 2A–D. Successive leftward shifts for strata of SNPs with higher significance in IBD and CD indicated that the proportion of MSA-associated SNPs increased considerably with higher levels of association with IBD and CD (and vice versa), suggesting significant shared genetic background between MSA and both IBD and CD (Fig. 2). In contrast, there was weak enrichment observed for other analyzed phenotypes (Fig. S1). We also did not observe significant (*P* < 0.05) genetic correlation between MSA and any of the 7 analyzed autoimmune disorders, which is likely because of the limited sample sizes of the MSA GWAS.

### Shared Loci Between MSA and IBD

**FIG. 3.**
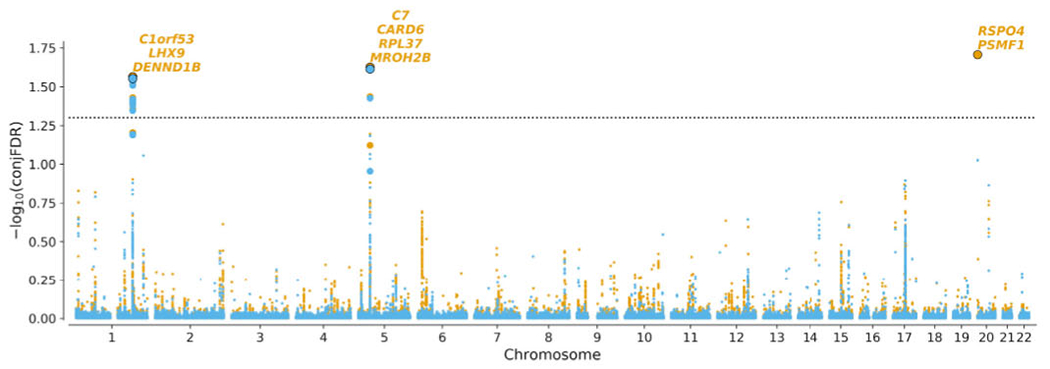
Manhattan plot of −log_10_(conjFDR) for MSA and CD (orange)/IBD (blue). Horizontal dashed black line shows the significance threshold conjFDR = 0.05. For each significant locus, genes within 100 kb of the locus lead SNP are shown. Lead variants at each locus are shown as bold dots with black border. Variants in high LD (*r*^2^ > 0.6) with the lead variant are shown as bold dots without border. [Color figure can be viewed at wileyonlinelibrary.com]

Distinct genetic overlap observed in the conditional Q-Q plot (Fig. 2) led us to perform genome-wide association screening by combining MSA with IBD and CD GWAS summary statistics in conjFDR analysis. Three LD-independent regions significantly associated with MSA and IBD/CD were identified at conjFDR < 0.05 (Table 1). Manhattan plot for conjFDR results is presented in Figure 3. Detailed regional association plots for identified loci are presented in Figure S2.

### Functional Annotation of Identified Loci

Functional annotation of 3 leading variants from loci shared between MSA and IBD/CD as identified in conjFDR analysis (conjFDR < 0.05) showed that 1 variant (rs4957144) is in the first intron of the *C7* gene, whereas 2 others, rs12740041 and rs116843836, are intergenic variants located upstream of the corresponding *DENND1B* and *RSPO4* genes (Table 1). The lead variant of the locus shared between MSA and IBD/CD on chromosome 5 at 5p13.1 (rs4957144, CADD, 14.2) has a CADD score above 12.37, suggesting deleteriousness^38^ (Table S1). Querying these 3 variants for eQTL status in the GTEx data^39^ revealed *DENND1B* (rs12740041), *TTC33* (rs4957144), and *PSMF1* (rs116843836) as potential target genes in various tissues (Table S7). Additional scan of brain and blood eQTL summary statistics with large sample sizes suggested rs12740041 as eQTL for *DENND1B* and rs4957144 as eQTL from *TTC33* in both blood and brain, whereas rs4957144 was also highlighted as potential eQTL for *RPL37* in brain (Table S8). *TTC33* is 158 kb downstream of rs4957144 (Fig. S4) and thus is not shown in Table 1.

Assessing a single-variant of eQTL data is prone to false-positives.^40^ We applied the LocusCompare tool to refine our eQTL findings and check whether loci identified in conjFDR analysis colocalize with eQTL signal. Notable colocalization was observed for rs12740041, where a clump of variants in LD (*r*^2^ > 0.4) significantly associated with MSA, CD, and IBD in the conjFDR analysis also revealed substantial deregulation of *DENND1B* expression in the brain and in several other tissues (Fig. S5A–C). Observed colocalization of conjFDR and eQTL signals in multiple tissues suggests that deregulation of *DENND1B* expression is more likely to be involved in MSA/IBD/CD pathogenesis. Other loci identified in our analysis did not show strong evidence of colocalization events (Fig. S5D–J).

Eight protein coding genes within 100 kb of leading SNPs identified in the conjFDR analysis of MSA versus IBD/CD (Table 1) together with *TTC33* highlighted in the eQTL analysis (Table S7) were selected as candidate genes for downstream analyses.

### Gene-Level Association Analysis

The 9 selected candidate genes were assessed to estimate the cumulative impact of protein-coding variants observed in independent IBD/CD and MSA cohorts. The SKAT test applied to all protein-coding variants (no minor allele frequency threshold) identified *C7* to be significantly associated with IBD, CD, and MSA (p_MSA_, 1.10 × 10^−03^; p_CD_, 2.74 × 10^−05^; p_IBD_, 5.99 × 10^−05^), suggesting the importance of the genetic variability within *C7* for these diseases. Three other genes from the same locus *CARD6* (p_IBD_, 3.33 × 10^−02^), *RPL37* (p_MSA_, 8.79 × 10^−03^), and *TTC33* (p_CD_, 9.46 × 10^−03^) were nominally significant in IBD, MSA, and CD correspondingly but did not survive multiple-testing correction (Table S8).

### Expression Analysis of Candidate Genes in the Degenerating Midbrain of Transgenic MSA Mice

**FIG. 4.**
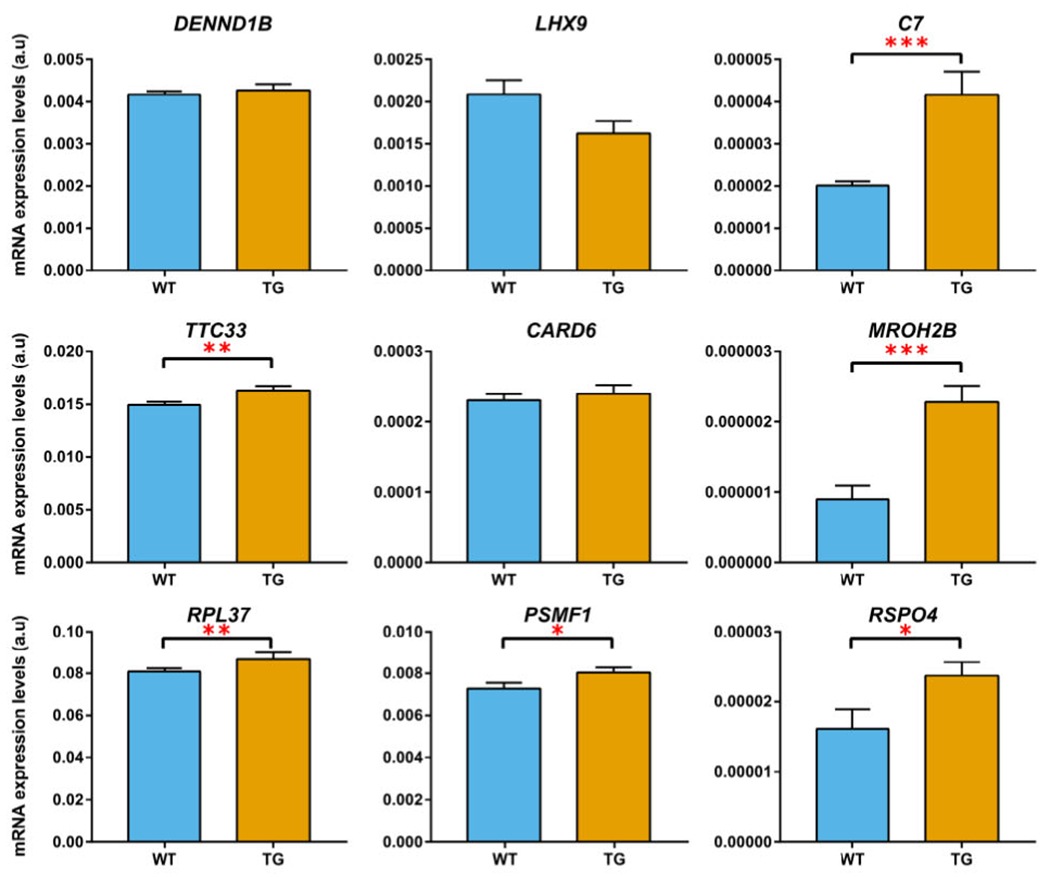
Comparison of expression in the midbrain between wild-type mice (blue bars) and transgenic MSA mice (orange bars) for candidate genes identified in the conjFDR and eQTL analyses. Genes with significantly different expression between wild-type and transgenic mice are marked either with as * (nominal *P* < 0.05), ** (nominal *P* < 0.01), or *** (nominal *P* < 0.005). *C7* and *MROH2B* genes survive multiple testing with Bonferroni-Dunn correction (adjusted *P* < 0.05). Error bars show standard error of the mean. [Color figure can be viewed at wileyonlinelibrary.com]

The PLP-hαSyn transgenic model is characterized by MSA-P-like striatonigral degeneration triggered by human α-synuclein overexpression in oligodendrocytes and partly mediated by neuroinflammatory responses.^35^ We assessed the expression of the 9 candidate genes as identified in conjFDR and eQTL analyses. *MROH2B* (*P* = 0.03) and *C7* (*P* = 0.04) showed significant dysregulation in the midbrain of MSA mice following multiple *t*-test comparisons corrected with the Bonferroni-Dunn method (Fig. 4, Table S5). However, the expression of *MROH2B* was negligible; therefore, we concluded that among the examined genes, *C7* is the top candidate linked to MSA-like neurodegeneration in the transgenic mouse model.

### Identification of Relevant Tissues and Cell Types

We identified elevated enrichment for blood/immune and digestive categories in the Roadmap data set (nominal *P* < 0.001; Fig. S3A, Table S2). Furthermore, the ImmGen data set highlighted a trend for enrichment for B and myeloid cells in MSA (nominal *P* < 0.01). Taken together, these results suggested the relevance of the immune and digestive systems in MSA pathogenesis. Although we observed a trend toward enrichment for various categories, they were not significant when corrected for multiple testing (FDR corrected *P* > 0.1), most likely because of the small sample size of our MSA cohort (Fig. S3).

### Gene Set Enrichment Analysis

Pathway analysis using DEPICT showed that the *ITGA2* subnetwork was the most significant gene set for variants shared between MSA and IBD/CD (nominal *P* = 5.55 × 10^−07^) as identified in conjFDR analysis with relaxed significance threshold (conjFDR < 0.35). However, it did not survive multiple-testing correction at FDR < 0.05 (Table S4).

## Discussion

The genetic studies so far failed to provide MSA-associated genetic loci.^13,19^ Nevertheless, because of clinical overlap with PD, a number of studies have assessed the association of top PD-associated loci with MSA.^41^ Thus far, results on the underlying genetic heterogeneity in MSA pathogenesis have been conflicting.^19^ The present study provides evidence of shared genetic etiology between IBD/CD and MSA, using a genome-wide genetic pleiotropy-informed approach.

Genetic-pleiotropy informed approaches have been successfully applied to identify shared loci for many complex diseases, including AD and PD.^42^ Previously published study showed substantial genetic overlap between PD and autoimmune diseases, specifically with CD and diabetes mellitus type 1.^4^ These studies highlight the involvement of the immune component in neurodegenerative diseases. Given that MSA is a synucleinopathy, here we investigated the extent of the shared genetic etiology between MSA and autoimmune diseases. Conditional Q-Q plots (Fig. 2) suggested a genetic overlap between MSA and IBD/CD. Our approach helped to expand the genetic spectrum of MSA pathogenesis. These putative loci were not reported in the previously published MSA GWAS and highlight the utility of such agnostic approaches in gene discovery.^19^

MSA GWAS data have yielded negligible heritability estimates, in a range of 2%–6%.^13^ Novel strategies should be applied to define new loci for rare neurodegenerative diseases such as MSA. A recently published study highlighted the relevance of using tissue- and cell-specific data in conjunction with GWAS data to identify loci that hitherto could not be identified using a GWAS approach alone.^36^ Furthermore, as it has been shown in various other complex diseases, including neurodegenerative diseases, simultaneous inclusion of common and rare variants increases the power to detect the underlying genetic underpinnings for complex diseases.^43^ By leveraging various gene expression data sets, our study showed that MSA heritability is enriched in genes related to blood and digestive categories in GTEx and Roadmap data sets as well as in genes showing elevated expression in immune cells (Fig. S3). Although the enrichment was not statistically significant, which is likely because of the small-sample MSA GWAS.

Functional annotation highlighted a locus on chromosome 5 at 5p13.1, which contains the *C7* gene and shows high deleterious and moderate regulatory scores (Table S1), indicating that genetic variability within *C7* could modulate MSA and IBD disease risks. The role of *C7* was strengthened by detecting its significant enrichment with common and rare variants in both MSA and IBD patients (Table S8). In addition, a PLP-hαSyn transgenic model of MSA-P-like neurodegeneration showed significant disturbance of *C7* expression in the midbrain (Fig. 4), a region specifically characterized by early neuroinflammatory response and neuronal loss linked to oligodendroglial α-synucleinopathy.^35^ This converging evidence suggests the involvement of *C7* in MSA pathogenesis, which will necessitate further clarification by detailed functional analyses. It is worth noting that we observed opposite directionality of the effects for *C7* in MSA and IBD/CD (Table 1), suggesting distinct mechanisms for both phenotypes. Our results should be considered in lieu of the findings that complex diseases often show substantial evidence of genetic pleiotropy,^44^ and it is likely that *C7* may exert different pathogenetic mechanims in MSA and IBD. Further functional studies are warranted to decipher the role of *C7* in MSA.

Our study reinforces the potential role of the “gut-brain axis” in neurodegenerative diseases, in particular, parkinsonian syndromes.^45^ For example, a previously published study showed considerable genetic overlap between PD, schizophrenia, and CD.^46^ Interestingly, some comorbidity between PD and CD was driven by the genetic variability within *LRRK2.*^47^ Furthermore, a recently published study further refined the *LRRK2* locus signal in CD and identified the p.*N2081D* variant driving increased CD risk.^47^ Of note, both p.*G2019S* and p.*N2081D* mutations are in the kinase domain of *LRRK2,* highlighting the role of kinase activity in disease pathogenesis.^47^ *LRRK2* has been shown to be involved in a diverse range of functions, including vesicular trafficking, autophagy, and inflammation.^48^

A recent population-based study from Denmark showed a 22% increased risk for PD for patients with IBD compared with non-IBD individuals. The authors also observed a trend, though nonsignificant, for increased risk for MSA in IBD patients.^49^ In our study, the *C7* gene showed increased burden of rare and common variants in both IBD and MSA cohorts (Table S8); however, the lead variant in this locus demonstrated opposite direction of effect in IBD/GD and MSA (Table 1). Further studies are required to assess clinical evaluation of IBD patients for MSA phenotype to discern the role of *C7* in IBD patients with the MSA phenotype.

Our study indicates a bidirectional mechanism between the central and enteric nervous system mediated via the gut microbiome, which is directly linked to the status of the intestinal immune system.^45^ Thus, the chronic state of inflammation can promote systemic inflammation and neuroinflammation.^50^ A recent study showed the migration of human α-synuclein after injecting into the intestinal wall of rats to the dorsal motor nucleus in the brain stem in a time-dependent manner, suggesting that changes induced in α-synuclein in the gut can directly affect the brain and elicit an immune response.^51^

The loci identified in our study are directly linked to immune-related activities. A recently published study identified genetic variability in the *C7* gene as a major risk factor for AD in the Han Chinese population.^52^ In addition, the complementary system has been shown to be implicated in a diverse range of functions, including amyloid-beta (Aβ) clearance, microglia activation, neuroinflammation, apoptosis, and neuron death.^53,54^ We observed notable colocalization of conjFDR association and eQTL signals for the *DENND1B* gene in brain and blood (Fig. S5). *DENND1B,* a member of the connecdenn family, has been shown to play a role in clathrin-mediated endocytosis.^55^ The evidence obtained from our study underlying the relevance of immune and vesicular trafficking is important for MSA pathogenesis. Our pathway analysis identified the *ITGA2* pathway as a “top hit” (Table S4). Integrins are cell adhesion mediators and have been shown to be involved in a diverse range of human diseases, including IBD, as has been shown in a recently published GWAS.^15^

Neuroinflammation characterized by microglial activation has been involved in neurodegenerative diseases, including MSA.^50,56^ Thus, interventional strategies targeting the neuroinflammation offers an alternative approach to either halt or slow disease progression. For example, a study by Mandler et al^51^ performed active immunization against α-synuclein in myelin basic protein MBP-α-Syn transgenic mice. They observed reduced α-synuclein colocalization in oligodendrocytes and astrocytes along with reduced neuronal death and motor deficits. Similarly, therapeutic strategies, which interfere with the synthesis of tumor necrosis factor-α, have been tested in animal models of PD,^51^ although results generated so far have been remained inconclusive.

Although power for detection of genome-wide significant signals was limited, our findings are corroborated by evidence from transgenic MSA models and independent exome cohorts. The current results might have potential clinical implications. More extensive clinical evaluation of patients with IBD/CD for monitoring immune/inflammatory and MSA-related symptoms should he allowed, as has been suggested in a recently published study.^49^

In conclusion, our study extends the genetic architecture of MSA and provides evidence of shared genetic etiology with IBD. Importantly, our findings extend the gut-brain axis spectrum from PD to atypical parkinsonian syndromes. ■

## Supplementary Material

Appendix s1

Tables

## Figures and Tables

**FIG. 1.**
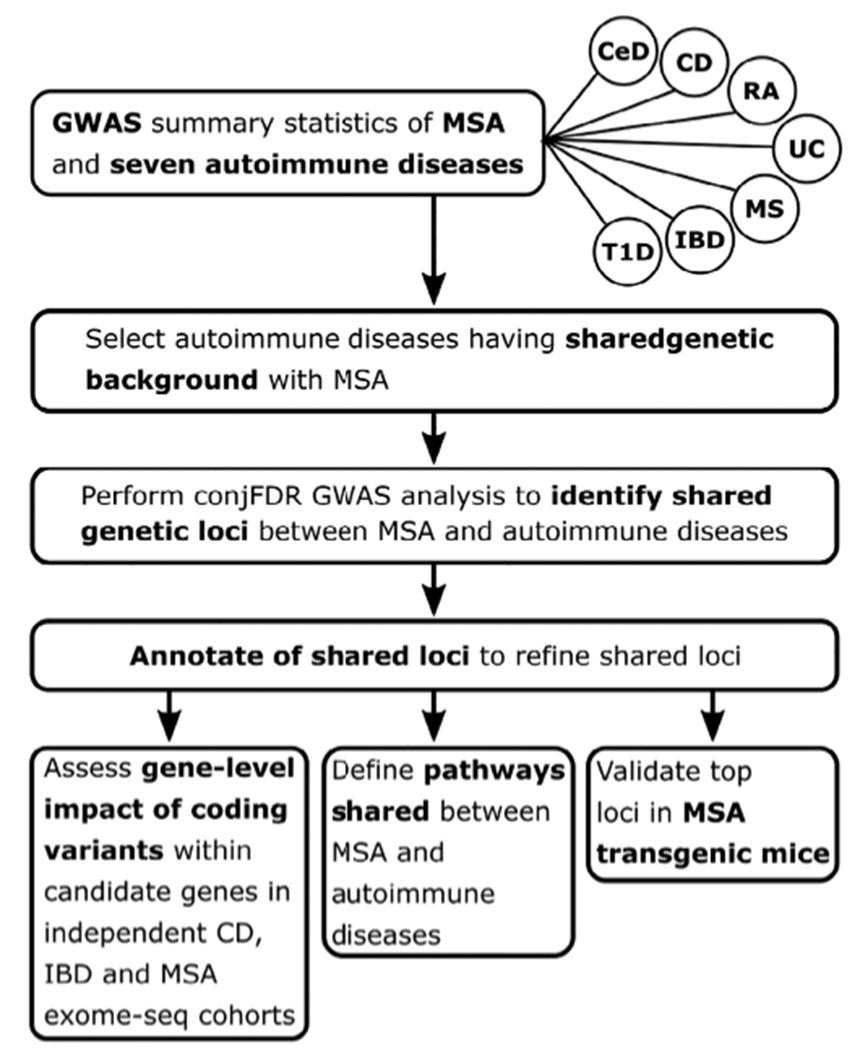
Flowchart representing major steps of the study. MSA, multiple system atrophy; CD, Crohn’s disease; IBD, inflammatory bowel disease, including CD, ulcerative colitis, and unclassified IBD cases; UC, ulcerative colitis; T1D, diabetes mellitus type 1; CeD, celiac disease; RA, rheumatoid arthritis; MS, multiple sclerosis.

**TABLE 1.** Significant (conjFDR < 0.05) loci shared between MSA and IBD/CD

Lead SNP	Chr region	Position	conjFDR of MSA and		Genes within 100 kb	Location relative to closest gene	Effect Size			*P*		
			CD	IBD			MSA	CD	IBD	MSA	CD	IBD
rs12740041	1q31.3	197,814,607	**2.73 × 10^−2^**	**2.80 × 10^−2^**	***C1orf53*** *LHX9* *DENND1B*	Upstream	−2.84 × 10^−1^	9.74 × 10^−2^	8.02 × 10^−2^	3.68 × 10^−5^	4.78 × 10^−7^	9.95 × 10^−8^
rs4957144	5p13.1	40,914,326	**2.37 × 10^−2^**	**2.44 × 10^−2^**	***C7*** *CARD6* *RPL37* *MR0H2B*	Intronic	2.82 × 10^−1^	−9.59 × 10^−2^	−6.46 × 10^−2^	3.14 × 10^−5^	2.90 × 10^−9^	2.56 × 10^−7^
rs116843836	20p13	1,033,414	**1.96 × 10^−2^**	9.42 × 10^−2^	***RSP04*** *PSMF1*	Upstream	−8.75 × 10^−1^	−2.08 × 10^−1^	−1.46 × 10^−1^	2.46 × 10^−5^	5.40 × 10^−5^	2.96 × 10^−4^

Chromosome (Chr) and position are indicated according to GRCh37. Significant conjFDR values (conjFDR < 0.05) are shown In boldface type. Closest gene is shown in bold. Effect size is given as beta regression coefficient from the original GWAS, and corresponding *P* values are shown without genomic correction.
