# Supplementary material for: Shared Genetics of Multiple System Atrophy and Inflammatory Bowel Disease
Source: Mov Disord. Author manuscript; Available in PMC 2022 Apr 6. (PMC8985479; doi:10.1002/mds.28338)
Supplement: Appendix s1 [file NIHMS1789384-supplement-Appendix_s1.docx]

# Supplementary Figures

| 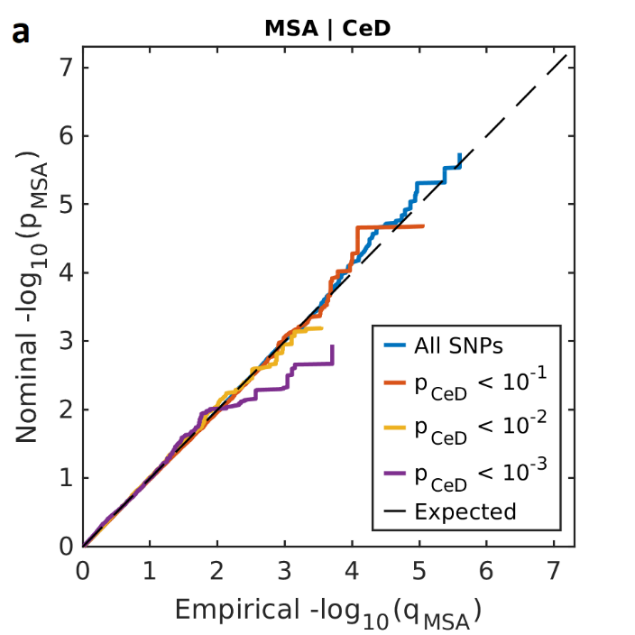 | 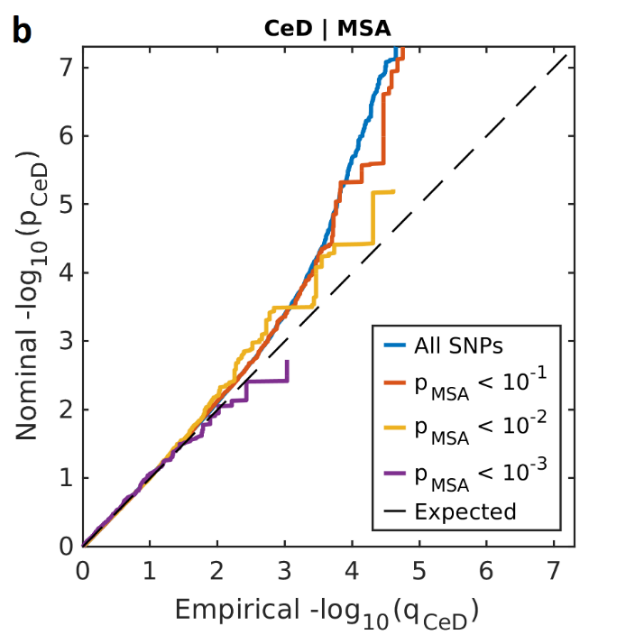 |
| --- | --- |
| 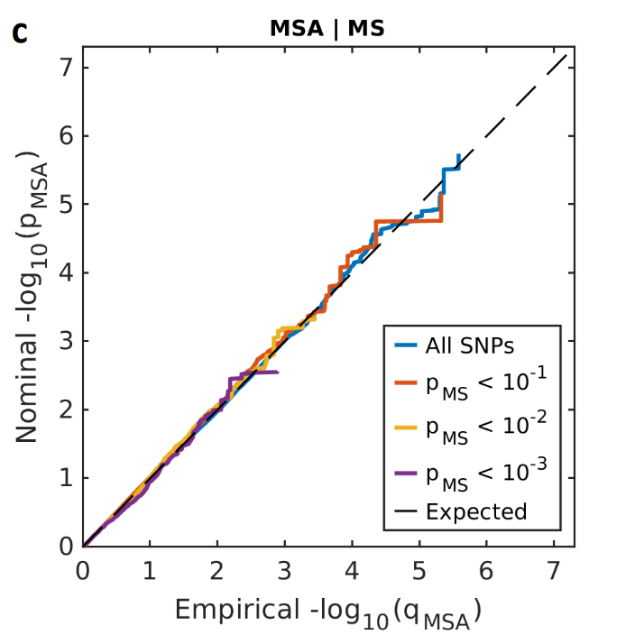 | 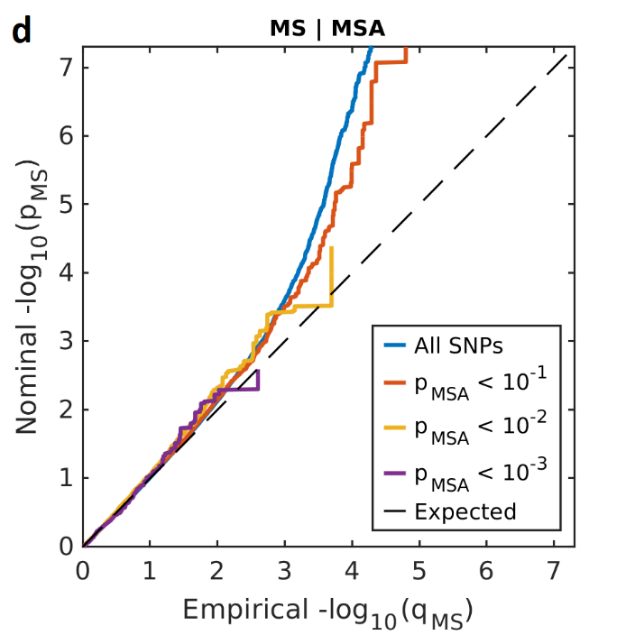 |
| 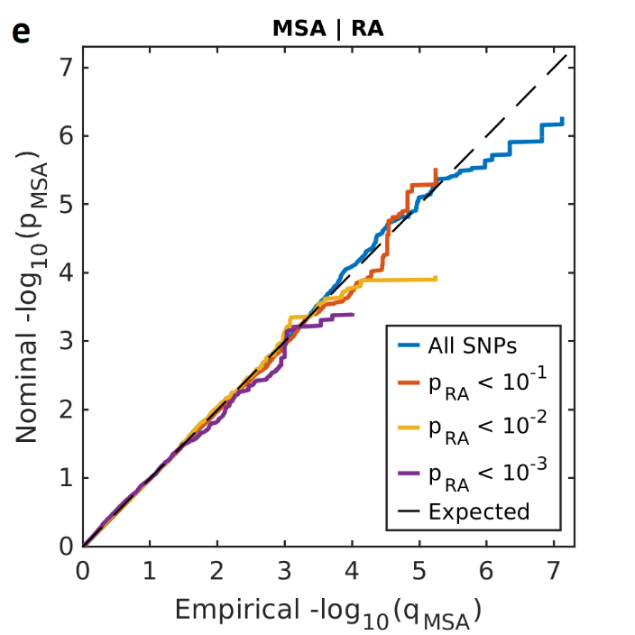 | 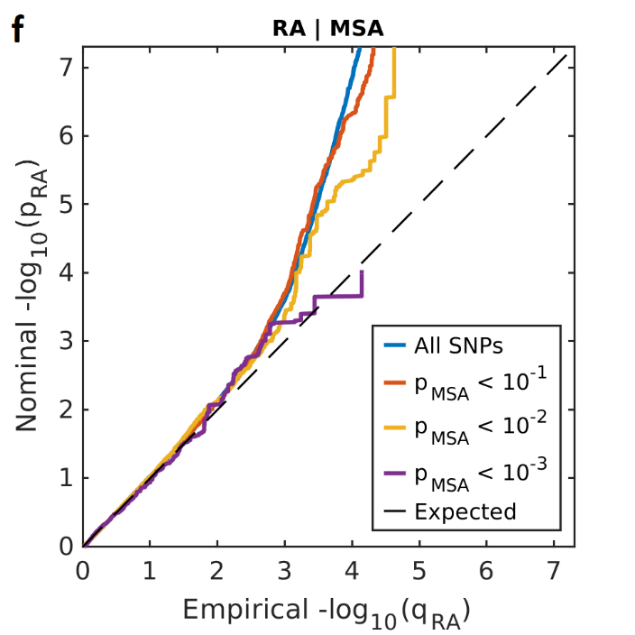 |
| 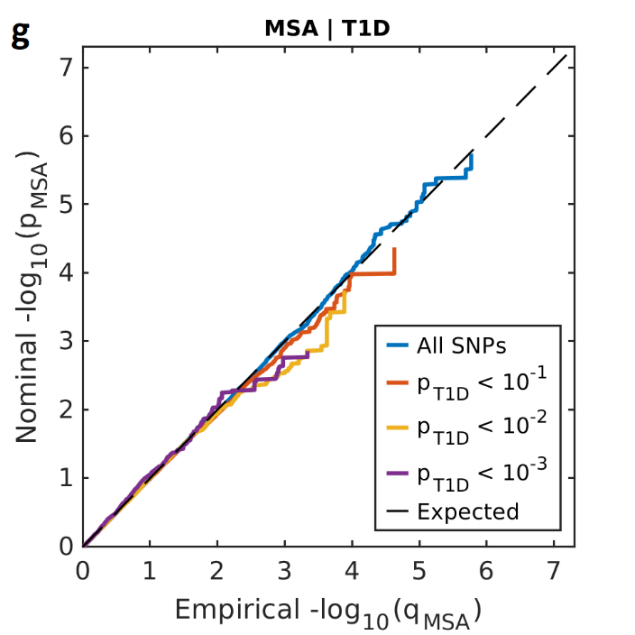 | 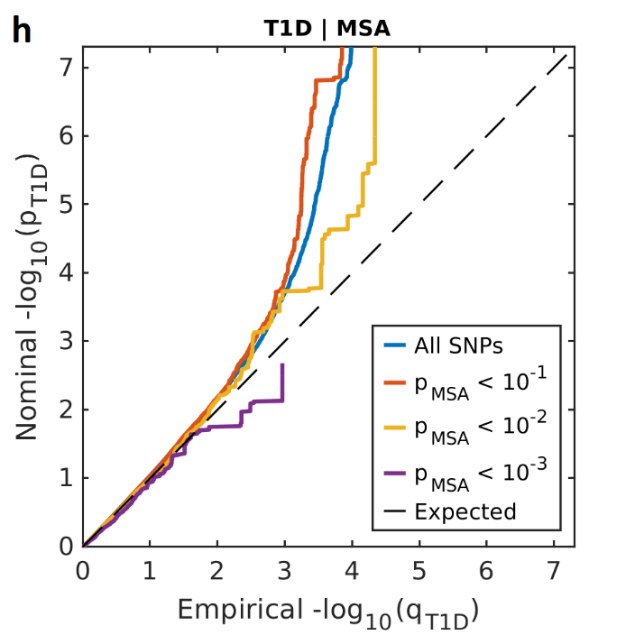 |
| 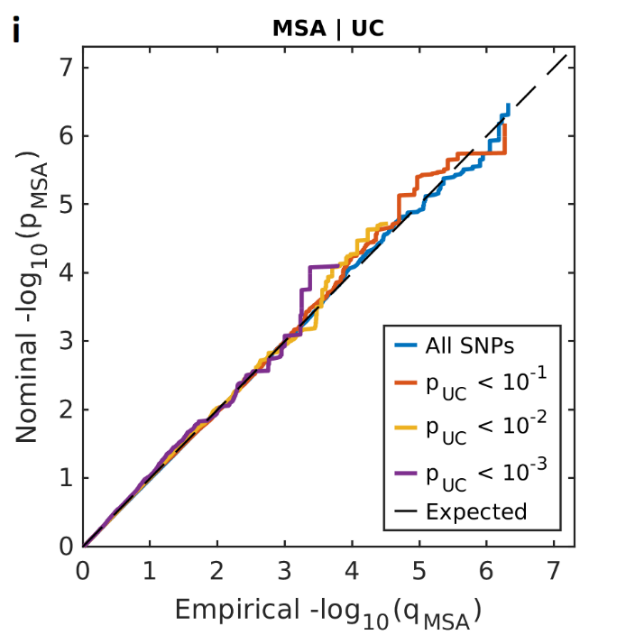 | 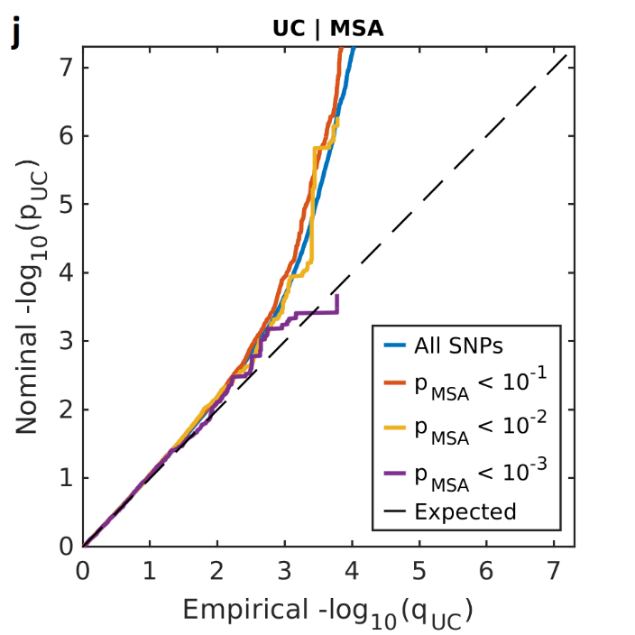 |
| **Supplementary Figure 1. Conditional Q-Q plot showing show relation between expected (x axis) and observed (y axis) signiﬁcance of SNPs in the primary phenotype when markers are stratiﬁed by their p-values in the conditional phenotype.** A sequence of 4 nested strata is presented: all SNPs (blue), p_conditional_phenotype_ < 0.1 (orange), p_conditional_phenotype_ < 0.01 (yellow) and p_conditional_phenotype_ < 0.001 (purple). Dashed black line demonstrates expected behavior under no association. Increasing degree of leftward deflection from the no-association line for strata of SNPs with higher significance in the conditional phenotype indicates polygenic overlap.  a: MSA conditioned on celiac disease (CeD); b: CeD conditioned on MSA; c: MSA conditioned on multiple sclerosis (MS); d: MS conditioned on MSA; e: MSA conditioned on rheumatoid arthritis (RA); f: RA conditioned on MSA; g: MSA conditioned on diabetes mellitus type 1 (T1D); h: T1D conditioned on MSA; i: MSA conditioned on ulcerative colitis (UC); j: UC conditioned on MSA. | |

| 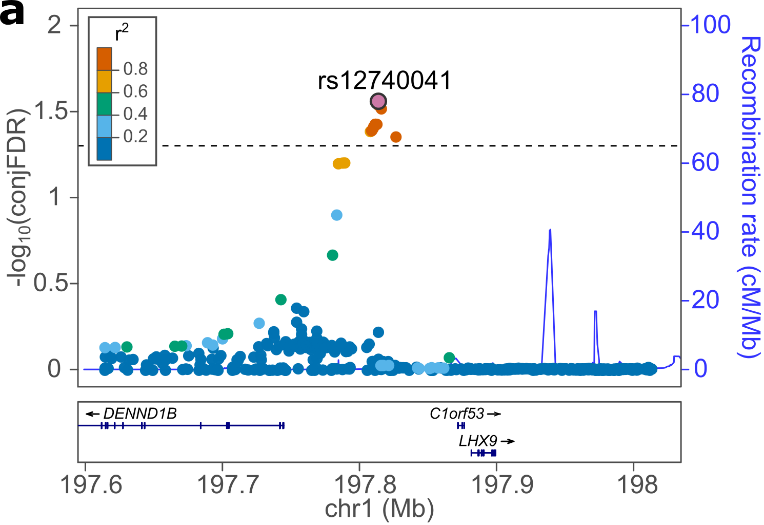 |
| --- |
| 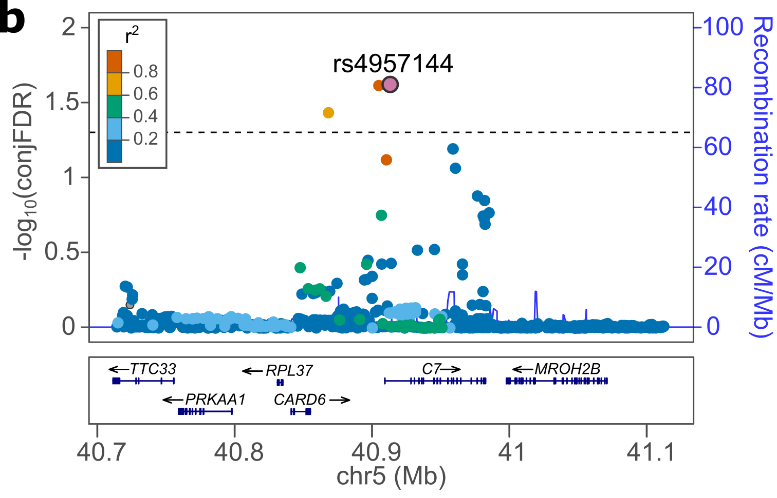 |
| 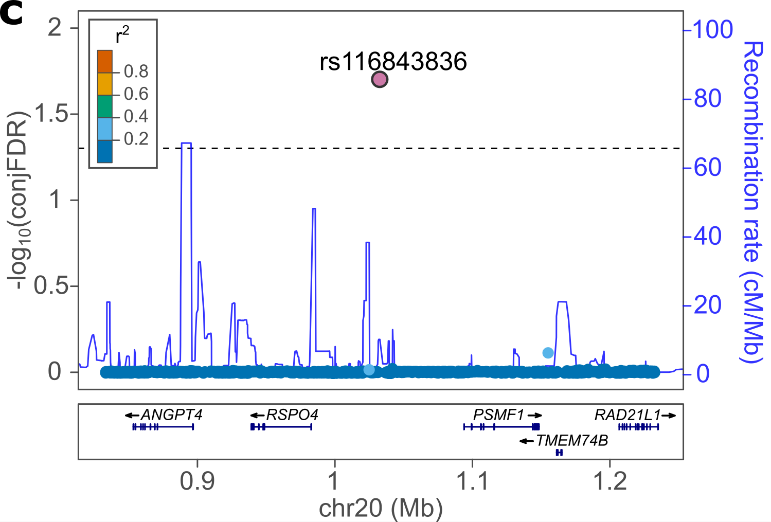 |
| **Supplementary Figure 2. Genetic context of loci identified in the conjFDR analysis.** The x-axis represents the positional location of the variants on the chromosome, left y-axis shows -log_10_(conjFDR) value if the variants. In each subplot, a variant with the strongest association is shown in the large purple circle. The color of the remaining markers reflects the degree of linkage disequilibrium (LD), with the strongest-associated variant measured as r^2^ coefficient (described in the legend). The recombination rate is plotted as a solid blue line; its value in centimorgan/megabase (cM/Mb) is indicated on the right y-axis. The black dashed lines indicate the conjFDR threshold = 0.05. Surrounding of the loci with the strongest signal at: a: rs12740041 (conjFDR=2.73E-02); b: rs4957144 (conjFDR=2.37E-02); c: rs116843836 (conjFDR=1.96E-02). |

| 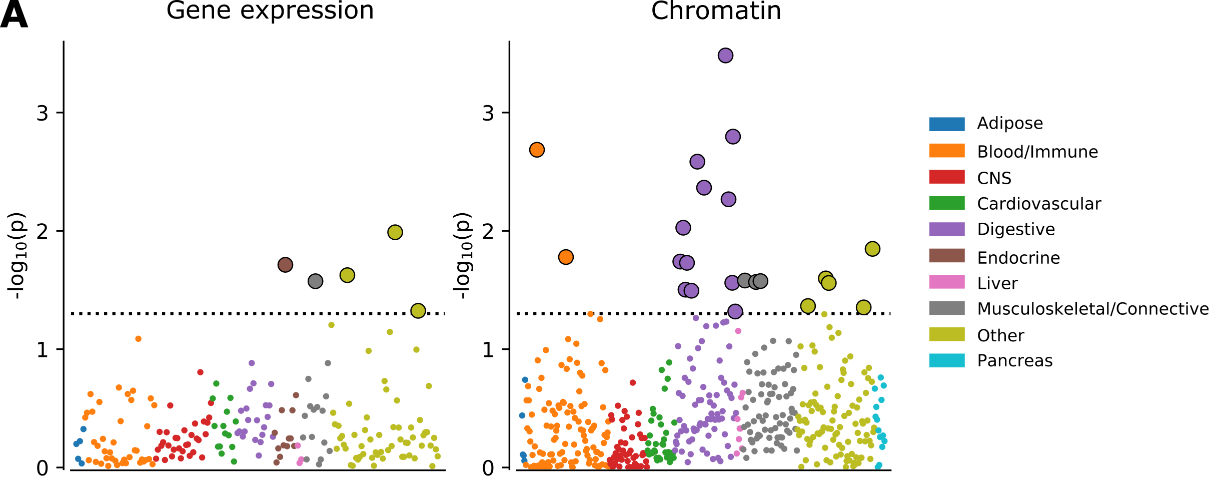 |
| --- |
| 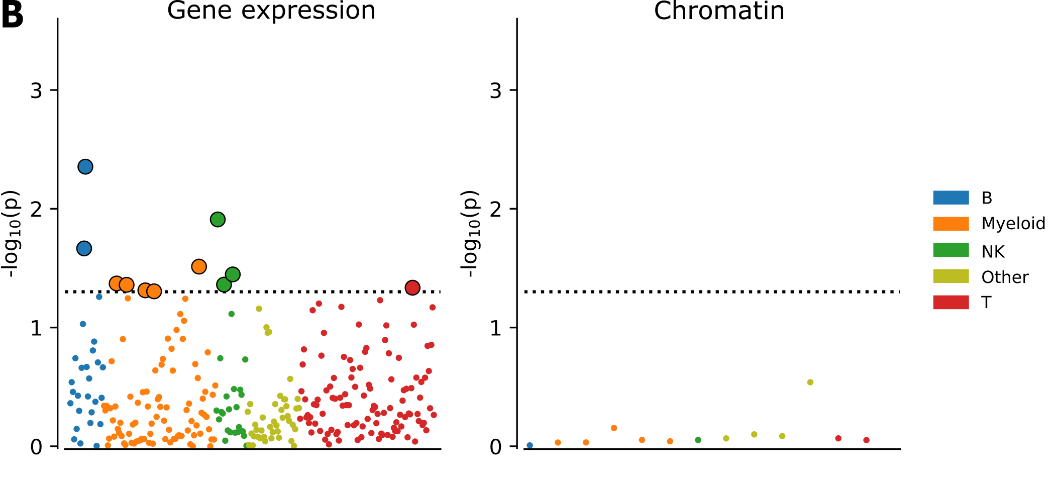 |
| 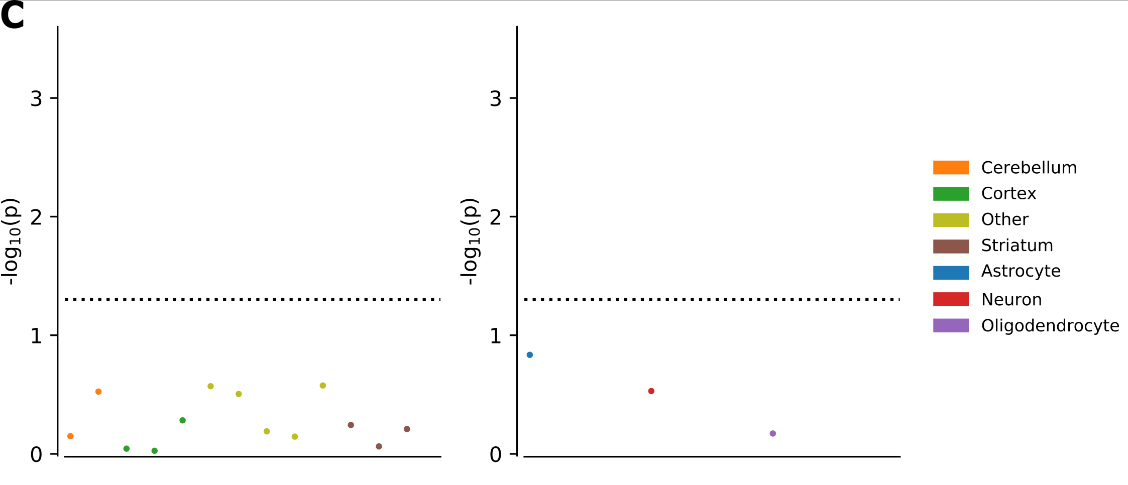 |
| **Supplementary Figure 3. Tissue/cell-type specific enrichment of MSA heritability.** Y-axis shows -log10 uncorrected p-value. None of tissue/cell type is significant after FDR correction with 0.05 threshold. Nominally significant results (p < 0.05) are plotted with larger outlined circles. Horizontal dashed line represents nominal significance threshold -log_10_(0.05). Numerical results are presented in Supplementary Table 2. A: Multiple tissue analysis using gene expression data from GTEx and Franke et al. and chromatin data from Roadmap and EN-TEx datasets (top hit in Digestive category has FDR = 0.16). B: Immune cell type analysis using expression data from ImmGen and chromatin data from Corces et al. C: Results for different brain regions from GTEx (left), and three brain cell types from Cahoy et al. (right). |

| 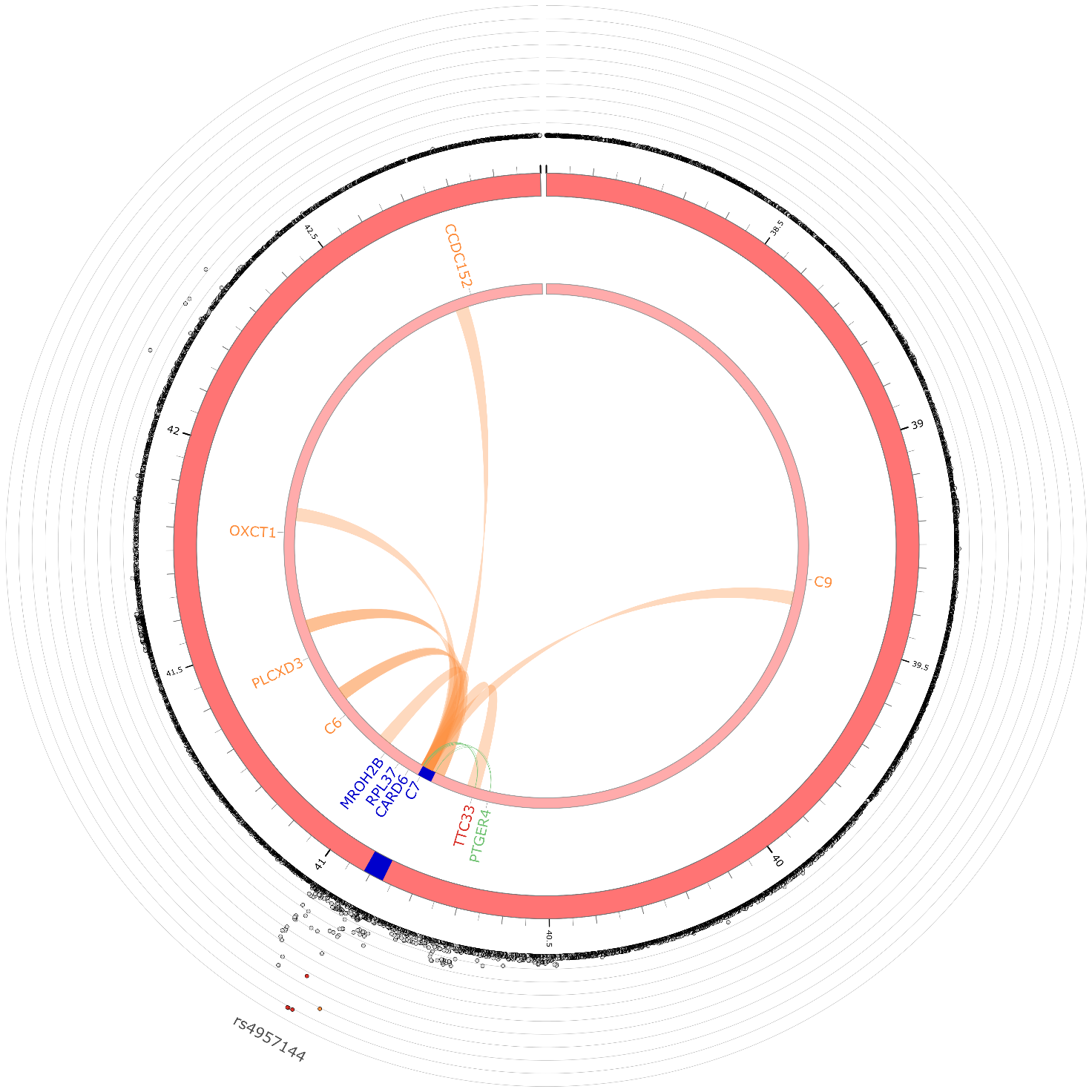 |
| --- |
| **Supplementary Figure 4. Circos plot demonstrating locus on chromosome 5 at 5p13.1 identified in the conjFDR analysis (Table 1).** Lead SNP is labeled at the outer circle. Genes within 100 kb of the lead SNP have blue labels. Significant eQTL target genes of significant SNPs in the locus have green labels and chromatin interaction regions are showed with orange. The gene has res label if it is both a significant eQTL target and is in the chromatin interaction region. |

| 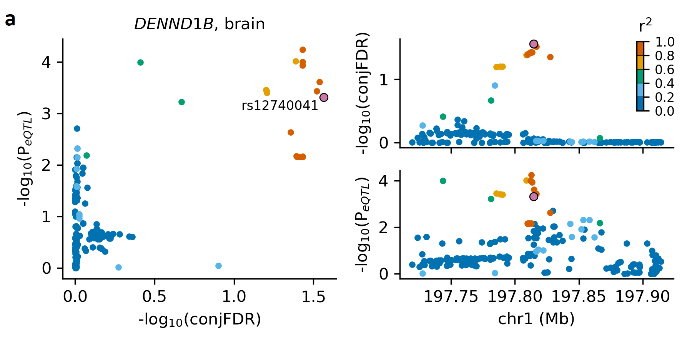 | 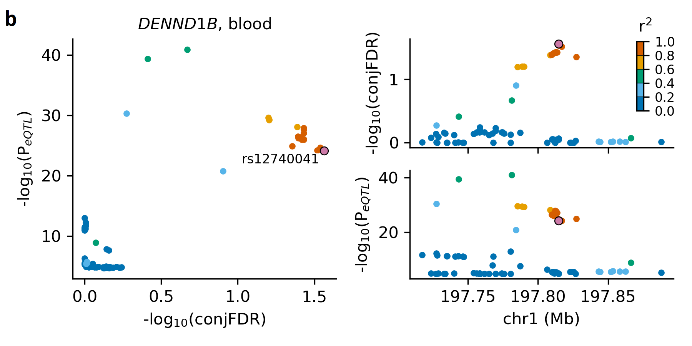 |
| --- | --- |
| 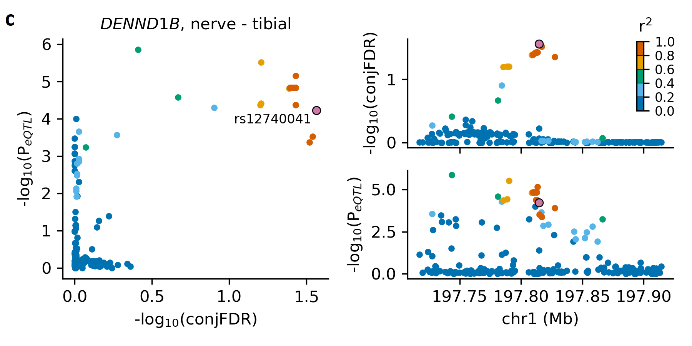 | 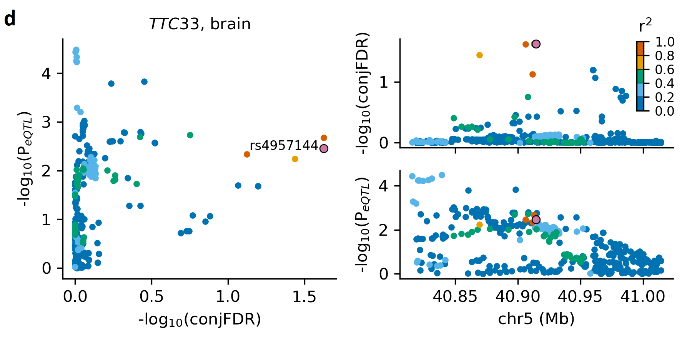 |
| 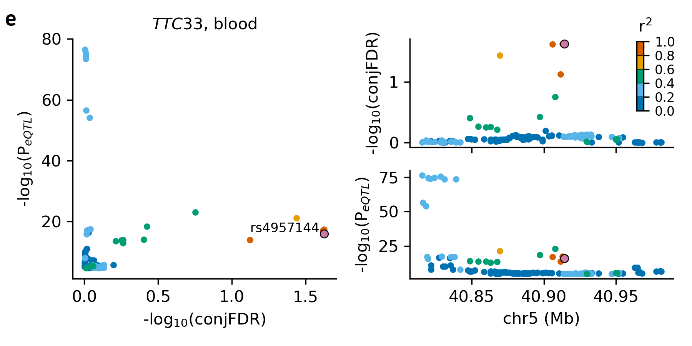 | 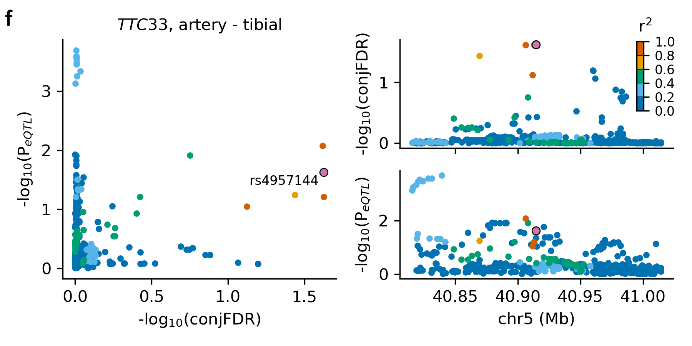 |
| 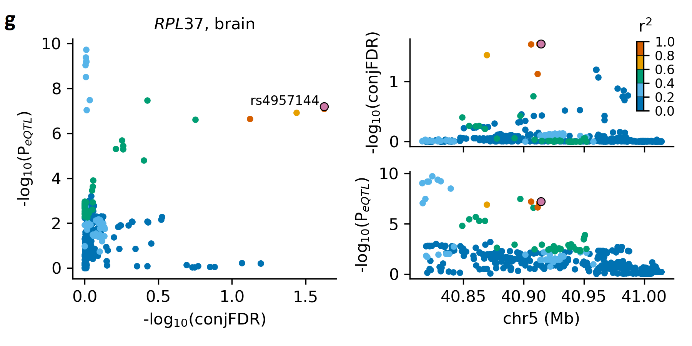 | 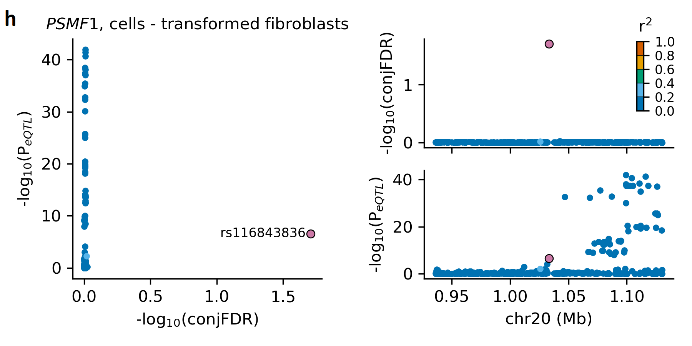 |
| 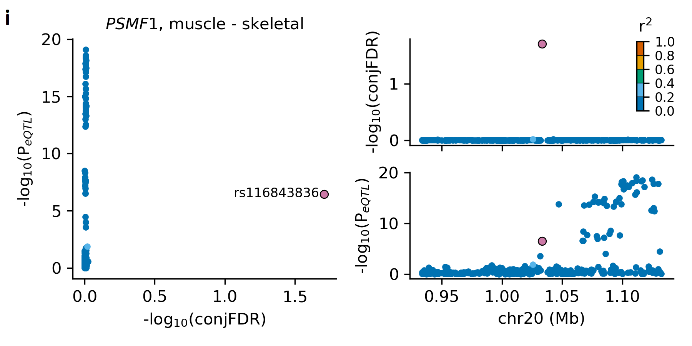 | 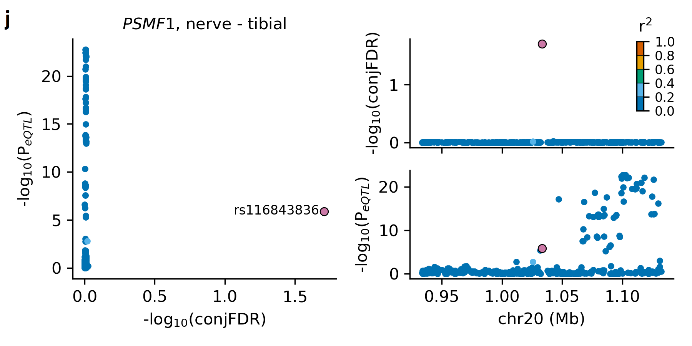 |
| **Supplementary Figure 5. Colocalization of association signals from conjFDR analysis and eQTL data from multiple tissues for genes within loci identified in conjFDR analysis.** Scatter plot of the -log_10_(conjFDR) plotted against -log_10_(P_eQTL_) (left), regional association plot for -log_10_(conjFDR) values (top right), regional association plot for -log_10_(P_eQTL_) (bottom right). In each subfigure lead variant of the locus identified in conjFDR analysis is shown in the large purple circle and labeled in the scatterplot. Other variants are colored according to the degree of linkage disequilibrium (LD, measured as r^2^ coefficient) with corresponding lead variant. For each subplot (a - j) the title of the scatterplot shows corresponding gene name and tissue, where brain stands for the eQTL data from Qi et al.^1^, blood refers to the eQTL data from Võsa et al.^2^ and the rest represents various tissues from GTEx. | |

# Supplementary Tables

Supplementary tables 1 – 8 are in the separate Excel file.

# Supplementary Methods

## Shared loci between MSA and IBD

The phenotypes which showed substantial genetic overlap with MSA in conditional Q-Q plots were further analyzed with genome-wide conjFDR method^3, 4^ (see URLs) to identify shared genetic loci between MSA and autoimmune diseases. The conjFDR method is based on the condFDR approach. The condFDR method combines summary statistics from two phenotypes (primary and conditional) and estimates a posterior probability that a variant has no association in the primary phenotype, given that p-values of the variant in both primary and conditional phenotypes are lower than observed p-values. Therefore, condFDR may boost the discovery of loci associated with a primary phenotype by leveraging associations with conditional phenotype. The increase in statistical power is achieved by re-ranking variants as compared to ranking based on the original GWAS p-values^3^. On the contrary, the ranking induced by the standard unconditional FDR (e.g. Benjamini–Hochberg procedure) does not change the order of variants as compared to nominal p-values.

The conjFDR is an extension of condFDR allowing identification of loci associated with both phenotypes simultaneously. For each specific variant, conjFDR is defined as a maximum of two condFDR values (taking one phenotype as primary and another as conditional and then swapping their roles). Therefore, conjFDR provides a conservative estimate of a posterior probability that a variant has no association with either of the phenotypes, given that the p-values for that variant in both analyzed phenotypes are lower than the observed p-values. For more technical details please refer to the section below and the original publication^3^.

## Conditional/Conjunctional FDR analyses

The following brief description of conditional/conjunctional false discovery rate (condFDR/conjFDR) method is based on the papers^3, 5^ where the method was introduced and subsequent correction to the second paper^6^.

To explain the condFDR/conjFDR method, we will first review the concept of standard (unconditional) false discovery rate (FDR). Then we describe the condFDR method. The latter is an extension of the standard FDR, which incorporates information from genome-wide association summary statistics of a second phenotype to adjust its significance level. After that we will present a formal definition of conjFDR for two phenotypes and show that it can be estimated as a maximum of two conditional FDRs.

In empirical Bayes interpretation, for a given p-value cutoff, FDR can be defined as follows ^7^:

$$FDR\left( p \right)=\frac{\pi_{0}F_{0}(p)}{F(p)}$$

where $\pi_{0}$ is the *a priori* fraction of null SNPs, $F_{0}$ is the null cumulative distribution function (CDF), and $F$ is the CDF of all SNPs, both null and non-null. Under the null hypothesis, $F_{0}$ is the CDF of the uniform distribution on the unit interval [0,1], so $F_{0}\left( p \right)=p$ and the latter formula reduces to: $FDR\left( p \right)={\pi_{0}p}/{F(p)}$. Having this definition, the conditional FDR for two phenotypes can be defined as the posterior probability that a given SNP is null for the first phenotype given that the p-values for both phenotypes are as small as or smaller than the observed p-values. Formally this can be expressed as:

$$CondFDR\left( p_{1},p_{2} \right)=\frac{\pi_{0}(p_{2})p_{1}}{F\left( p_{1} | p_{2} \right)}$$

where $p_{1}$ and $p_{2}$ are p-values of SNPs in the first and the second phenotypes correspondingly, $\pi_{0}(p_{2})$ is the conditional proportion of null SNPs and $F\left( p_{1} | p_{2} \right)$ is the conditional cdf for the first phenotype given that p-values for the second phenotype are $p_{2}$ or smaller. We denote the conditional FDR for phenotype 1 (pt1) given phenotype 2 (pt2) as ${FDR}_{pt1|pt2}$. In our calculations, we produce a conservative estimate of ${FDR}_{pt1|pt2}$ by setting $\pi_{0}\left( p_{2} \right)=1$ and using the empirical conditional cdf in place of $F\left( p_{1} | p_{2} \right)$.

In our study, conjFDR is used to identify SNPs that are associated with two phenotypes simultaneously. It is defined as the posterior probability that a given SNP is null **for either phenotype or** both phenotypes simultaneously when the p-values for both phenotypes are as small or smaller than the observed p-values. Formally, the conjunctional FDR is defined as:

$$ConjFDR\left( p_{1},p_{2} \right)= \frac{\pi_{0}F_{0}(p_{1},p_{2})}{F(p_{1},p_{2})}+\frac{\pi_{1}F_{1}(p_{1},p_{2})}{F(p_{1},p_{2})}+\frac{\pi_{2}F_{2}(p_{1},p_{2})}{F(p_{1},p_{2})}$$

where $\pi_{0}$ is the *a priori* fraction of SNPs null for both phenotypes simultaneously, $F_{0}(p_{1},p_{2})$ is the joint null cdf, $\pi_{1}$ is the *a priori* fraction of SNPs non-null for the pt1 and null for pt2 with $F_{1}(p_{1},p_{2})$ the joint cdf of these SNPs, and $\pi_{2}$ is the *a priori* proportion of SNPs non-null for pt2 and null for pt1, with joint cdf $F_{2}(p_{1},p_{2})$. $F(p_{1},p_{2})$ is the joint overall mixture cdf for all SNPs of both phenotypes. We denote conjunctional FDR for phenotype 1 and phenotype 2 as ${FDR}_{pt1\&pt2}$.

A model-free conservative estimation of the conjunctional FDR for phenotypes pt1 and pt2 can be calculated as:

$${FDR}_{pt1\&pt2}=max\left\{ {FDR}_{pt1|pt2},{FDR}_{pt2|pt1} \right\}$$

Using upwardly biased estimates of conditional FDRs ${FDR}_{pt1|pt2}$ and ${FDR}_{pt2|pt1}$ as described above and noting that for enriched samples, p-values will tend to be smaller than predicted from the uniform distribution (giving $F_{1}\left( p_{1} \right)\geq p_{1}$ and $F_{2}\left( p_{2} \right)\geq p_{2}$), the latter equation follows from:

$$max\left\{ {FDR}_{pt1|pt2},{FDR}_{pt2|pt1} \right\}=max\left\{ \frac{p_{1}F_{2}(p_{2})}{F(p_{1},p_{2})},\frac{p_{2}F_{1}(p_{1})}{F(p_{1},p_{2})} \right\}\geq\left( \pi_{0}+\pi_{1}+\pi_{2} \right)max\left\{ \frac{p_{1}F_{2}(p_{2})}{F(p_{1},p_{2})},\frac{p_{2}F_{1}(p_{1})}{F(p_{1},p_{2})} \right\}\geq{(\pi_{0}p_{1}p_{2}+{\pi_{1}p}_{2}F_{1}(p_{1})+{\pi_{2}p}_{1}F_{2}(p_{2}))}/{F(p_{1},p_{2})}$$

Assuming that SNPs are independent if one or both are null (that is reasonable for disjoint samples), the last quantity is exactly equal to the formal definition of $ConjFDR\left( p_{1},p_{2} \right)$ above.

## Gene-level association tests

The top loci from the conjFDR analysis were further evaluated to determine the role of common and rare variants in IBD/CD and MSA cohorts respectively.

An exome-wide chip covering a total of 205,313 SNPs was used to determine the role of common and rare variants in IBD/CD. RAREMETALWORKER was used with the first ten principal components from PCA as covariates to analyze individual CD and IBD studies and to generate association summary statistics^8^. The exome chip genotype data was converted into Variant Call Format (https://github.com/samtools/hts-specs) format with PLINK ^9^, and variants were functionally annotated with EPACTS (v3.2.3; <http://genome.sph.umich.edu/wiki/EPACTS>).

A two-step approach was followed: (i) SKAT test^10^ was performed in German CD cohort (exome array study including 4,989 cases and 16,307 controls, using the chip with 205,313 mostly rare coding variants) followed by replication (ii) in a combined IBD cohort (15,236 cases and 34,668 controls). The total number of genes covered in CD cohort was: n = 15,975 for all variants, n = 15,742 for rare variants, and in IBD cohort was: n = 16,306 for all variants, n = 16,138 for rare variants. SKAT test in both cohorts was conducted using all coding variants within identified genes (Table 3). P-values resulting from these tests were corrected for multiple testing using Bonferroni correction.

Whole exome sequencing data from 358 European-ancestry MSA patients and 1,297 neurologically healthy, European controls were generated using the SureSelect Exome target enrichment technology according to the manufacturer’s protocol (Agilent, CA, USA). Sequence alignment to the human reference genome (hg19) and variant calling were performed using the Genome Analysis Toolkit. After removal of duplicate samples using Picard software, stringent quality control filters were applied, including the removal of non-European ancestry individuals and cryptically related individuals, exclusion of individuals with the discrepancy between reported sex and genotypic sex and individuals with high genotype missingness or extreme heterozygosity. Next, all variants were annotated using ANNOVAR^11^. Principle components were generated using flashPCA. SKAT test was performed on all coding variants within identified genes using RVtests and corrected for multiple testing using Bonferroni correction (Table 3).

## Identification of relevant tissues and cell types

To understand whether conjFDR genetic-pleiotropy analyses help further to explain the heritability in MSA by identifying genes expressed in certain tissue/cell types, we used a modified LD score regression applied to specifically expressed genes (LD-SEG) method^12^. In brief, for each gene a t-test was evaluated for the tissue/cell type-specific expression level. Subsequently, the top 10% of genes were selected and a 100 kb window was added around the transcribed region of each selected gene. Finally, stratified LD score regression^13^ was applied to resulting genetic regions. This procedure was then repeated for each tissue/cell type of interest. We used publicly available tissue/cell type datasets together with corresponding precomputed gene sets and respective LD-scores as described in the LD-SEG method^12^. A total of seven datasets were used for analyses, including data from GTEx (53 tissues and 13 brain regions)^14^, Franke lab (152 tissues)^15, 16^, Roadmap Epigenomics consortium (396 tissues and cell types)^17^, EN-TEx (93 tissues and cell types), Cahoy et al.^18^ (3 brain cell types), ImmGen Consortium (292 immune cell types)^19^ and Corces at al.^20^ (13 cell types). The analysis was performed using the protocol described in the online documentation of LD-SEG (https://github.com/bulik/ldsc/wiki/Cell-type-specific-analyses).

## Gene set enrichment analysis

DEPICT (depict_140721)^15^ was applied to get biological insights from lead variants of loci shared between MSA and overlapping autoimmune diseases, as identified in the conjFDR analysis with relaxed significance threshold (conjFDR<0.35) (Supplementary Table 6). DEPICT is a phenotype-agnostic data-driven integrative method that employs reconstituted gene sets based on massive numbers of experiments measuring gene expression to (1) prioritize genes and pathways related to observed genetic associations and (2) highlight tissues where prioritized genes are highly expressed. Gene set enrichment analysis in DEPICT is based on a ‘guilt-by-association’ procedure^16^ which prioritizes genes that share predicted functions with genes from the other associated loci more often than expected by chance. Precomputed GWAS based on arbitrarily generated phenotypes is used to estimate the distribution of p-values under null expectation and to perform multiple testing correction by computing FDR.

In our analysis, we followed an analysis protocol as described in the online DEPICT documentation (<https://data.broadinstitute.org/mpg/depict/documentation.html>). For MSA and overlapping autoimmune disease summary statistic first variants were clumped with PLINK^9^ (using flags --clump-p1 1e-5 --clump-kb 500 --clump-r2 0.05). Resulting tag variants were analyzed with the standard DEPICT pipeline using an FDR threshold of 0.05. Leading variants of loci shared between MSA and autoimmune diseases were directly used in standard DEPICT pipeline (no clumping was performed since these variants represent LD-independent regions).

Additionally, gene set enrichment test implemented in FUMA^21^ was applied to genes located within 100kb from the lead SNPs of the loci shared between MSA and autoimmune diseases as identified by conjFDR analysis (conjFDR<0.05). The hypergeometric test was used to assess enrichment of input genes in gene sets obtained from MsigDB^22, 23^ (which integrates information from KEGG^24^, REACTOME^25^, Gene Ontology^26^ and other sources) taking 19,283 protein-coding genes as a background gene set. Multiple testing correction (per data source) was performed using the Benjamini–Hochberg procedure to control FDR at 5% level.

FUMA gene set enrichment analysis revealed significant overrepresentation of candidate genes in several biological processes (based on Gene Ontology classification) (Supplementary Table 3). Many of these genes were related to immune responses.

# Supplementary References

1. Qi T, Wu Y, Zeng J, et al. Identifying gene targets for brain-related traits using transcriptomic and methylomic data from blood. Nat Commun 2018;9(1):2282.

2. Võsa U, Claringbould A, Westra H-J, et al. Unraveling the polygenic architecture of complex traits using blood eQTL meta-analysis. bioRxiv 2018:447367.

3. Andreassen OA, Thompson WK, Schork AJ, et al. Improved detection of common variants associated with schizophrenia and bipolar disorder using pleiotropy-informed conditional false discovery rate. PLoS Genet 2013;9(4):e1003455.

4. Smeland OB, Frei O, Shadrin A, et al. Discovery of shared genomic loci using the conditional false discovery rate approach. Hum Genet 2019.

5. Andreassen OA, Djurovic S, Thompson WK, et al. Improved detection of common variants associated with schizophrenia by leveraging pleiotropy with cardiovascular-disease risk factors. Am J Hum Genet 2013;92(2):197-209.

6. Andreassen OA, Thompson WK, Schork AJ, et al. Correction: Improved Detection of Common Variants Associated with Schizophrenia and Bipolar Disorder Using Pleiotropy-Informed Conditional False Discovery Rate. PLoS Genet 2015;11(11):e1005544.

7. Efron B. Large-scale inference : empirical Bayes methods for estimation, testing, and prediction. Cambridge ; New York: Cambridge University Press, 2010.

8. Feng S, Liu D, Zhan X, Wing MK, Abecasis GR. RAREMETAL: fast and powerful meta-analysis for rare variants. Bioinformatics 2014;30(19):2828-2829.

9. Chang CC, Chow CC, Tellier LC, Vattikuti S, Purcell SM, Lee JJ. Second-generation PLINK: rising to the challenge of larger and richer datasets. Gigascience 2015;4:7.

10. Wu MC, Lee S, Cai T, Li Y, Boehnke M, Lin X. Rare-variant association testing for sequencing data with the sequence kernel association test. Am J Hum Genet 2011;89(1):82-93.

11. Wang K, Li M, Hakonarson H. ANNOVAR: functional annotation of genetic variants from high-throughput sequencing data. Nucleic Acids Res 2010;38(16):e164.

12. Finucane HK, Reshef YA, Anttila V, et al. Heritability enrichment of specifically expressed genes identifies disease-relevant tissues and cell types. Nat Genet 2018;50(4):621-629.

13. Finucane HK, Bulik-Sullivan B, Gusev A, et al. Partitioning heritability by functional annotation using genome-wide association summary statistics. Nat Genet 2015;47(11):1228-1235.

14. Consortium GT. The Genotype-Tissue Expression (GTEx) project. Nat Genet 2013;45(6):580-585.

15. Pers TH, Karjalainen JM, Chan Y, et al. Biological interpretation of genome-wide association studies using predicted gene functions. Nat Commun 2015;6:5890.

16. Fehrmann RS, Karjalainen JM, Krajewska M, et al. Gene expression analysis identifies global gene dosage sensitivity in cancer. Nat Genet 2015;47(2):115-125.

17. Roadmap Epigenomics Consortium, Kundaje A, Meuleman W, et al. Integrative analysis of 111 reference human epigenomes. Nature 2015;518(7539):317-330.

18. Cahoy JD, Emery B, Kaushal A, et al. A transcriptome database for astrocytes, neurons, and oligodendrocytes: a new resource for understanding brain development and function. J Neurosci 2008;28(1):264-278.

19. Heng TS, Painter MW, Immunological Genome Project C. The Immunological Genome Project: networks of gene expression in immune cells. Nat Immunol 2008;9(10):1091-1094.

20. Corces MR, Buenrostro JD, Wu B, et al. Lineage-specific and single-cell chromatin accessibility charts human hematopoiesis and leukemia evolution. Nat Genet 2016;48(10):1193-1203.

21. Watanabe K, Taskesen E, van Bochoven A, Posthuma D. Functional mapping and annotation of genetic associations with FUMA. Nat Commun 2017;8(1):1826.

22. Subramanian A, Tamayo P, Mootha VK, et al. Gene set enrichment analysis: a knowledge-based approach for interpreting genome-wide expression profiles. Proc Natl Acad Sci U S A 2005;102(43):15545-15550.

23. Liberzon A, Birger C, Thorvaldsdottir H, Ghandi M, Mesirov JP, Tamayo P. The Molecular Signatures Database (MSigDB) hallmark gene set collection. Cell Syst 2015;1(6):417-425.

24. Kanehisa M, Sato Y, Furumichi M, Morishima K, Tanabe M. New approach for understanding genome variations in KEGG. Nucleic Acids Res 2019;47(D1):D590-D595.

25. Fabregat A, Jupe S, Matthews L, et al. The Reactome Pathway Knowledgebase. Nucleic Acids Res 2018;46(D1):D649-D655.

26. Camon E, Magrane M, Barrell D, et al. The Gene Ontology Annotation (GOA) Database: sharing knowledge in Uniprot with Gene Ontology. Nucleic Acids Res 2004;32(Database issue):D262-266.
